# The Hippo transducers TAZ/YAP and their target CTGF in male breast cancer

**DOI:** 10.18632/oncotarget.9668

**Published:** 2016-05-27

**Authors:** Anna Di Benedetto, Marcella Mottolese, Francesca Sperati, Cristiana Ercolani, Luigi Di Lauro, Laura Pizzuti, Patrizia Vici, Irene Terrenato, Isabella Sperduti, Abeer M. Shaaban, Sreekumar Sundara-Rajan, Maddalena Barba, Valerie Speirs, Ruggero De Maria, Marcello Maugeri-Saccà

**Affiliations:** ^1^ Department of Pathology, “Regina Elena” National Cancer Institute, Rome, Italy; ^2^ Biostatistics-Scientific Direction, “Regina Elena” National Cancer Institute, Rome, Italy; ^3^ Division of Medical Oncology B, “Regina Elena” National Cancer Institute, Rome, Italy; ^4^ Department of Pathology, University Hospitals Birmingham NHS Foundation Trust, Birmingham, UK; ^5^ Leeds Institute of Cancer and Pathology, University of Leeds, Leeds, UK; ^6^ Scientific Direction, “Regina Elena” National Cancer Institute, Rome, Italy

**Keywords:** male breast cancer, Hippo pathway, Hippo transducers, TAZ, YAP

## Abstract

Male breast cancer (MBC) is a rare disease and its biology is poorly understood. Deregulated Hippo pathway promotes oncogenic functions in female breast cancer. We herein investigated the expression of the Hippo transducers TAZ/YAP and their target CTGF in MBC. Tissue microarrays containing samples from 255 MBC patients were immunostained for TAZ, YAP and CTGF. One hundred and twenty-nine patients were considered eligible. The Pearson's Chi-squared test of independence was used to test the association between categorical variables. The correlation between TAZ, YAP and CTGF was assessed with the Pearson's correlation coefficient. The Kaplan-Meier method and the log-rank test were used for estimating and comparing survival curves. Cox proportional regression models were built to identify variables impacting overall survival. Statistical tests were two-sided. Tumors were considered to harbor active TAZ/YAP-driven gene transcription when they co-expressed TAZ, or YAP, and CTGF. Patients whose tumors had the TAZ/CTGF and YAP/CTGF phenotypes experienced shorter overall survival compared with their negative counterparts (log rank *p* = 0.036 for both). TAZ/CTGF and YAP/CTGF tumors were associated with decreased survival in patients with invasive ductal carcinomas, G3 tumors, hormone receptor-positive tumors, and tumors with elevated Ki-67. Multivariate analyses confirmed that the TAZ/CTGF and YAP/CTGF phenotypes are independent predictors of survival (HR 2.03, 95% CI: 1.06–3.90, *p* = 0.033; and HR 2.00, 95% CI: 1.04–3.84, *p* = 0.037 respectively). Comparable results were obtained when excluding uncommon histotypes (TAZ/CTGF: HR 2.34, 95% CI: 1.16–4.73, *p* = 0.018. YAP/CTGF: HR 2.36, 95% CI: 1.17–4.77, *p* = 0.017). Overall, the TAZ/YAP-driven oncogenic program may be active in MBC, conferring poorer survival.

## INTRODUCTION

Male breast cancer (MBC) is one of the rarest cancers in men [1]. Even though its incidence is slowly rising [2], in Western countries only 0.5–1% of all breast cancers (BC) are diagnosed in men [1]. Its incidence rates rise linearly with age [1]. Risk factors include a number of diseases characterized by an abnormal estrogen-to-androgen ratio, such as obesity, liver diseases and Klinefelter's syndrome [3]. Germ-line mutations in BRCA1 and BRCA2 were also associated with MBC [4, 5].

MBC is a hormone-driven tumor and steroid receptors, namely the estrogen receptor (ER), progesterone receptor (PgR) and androgen receptor (AR) are often expressed [6, 7]. Consistently, hormonal therapies are central in the medical management of these patients, albeit studies reported so far described retrospective, small-sized case series [8–14]. Despite the apparent similarities between MBC and post-menopausal female breast cancer (FBC) [6], some studies highlighted the existence of gender-related molecular differences at the genomic [15–17], transcriptome [18, 19], and microRNA levels [20]. Nevertheless, characterization efforts of MBC are still in their infancy, and the complex molecular taxonomy of FBC needs to be carefully considered as a potential confounding factor in these comparisons. Overall, the nature of deregulated pathway nodes fuelling MBC remains largely elusive.

In FBC, overwhelming preclinical evidence showed that altered Hippo pathway feeds multiple oncogenic functions [21]. The Hippo signaling was originally found to be crucial during embryonic development [22]. In transgenic animal models its perturbation resulted in increased cell proliferation, decreased cell death, altered stem cell function and tumorigenesis [23–28]. Mammary gland defects were also observed upon manipulation of key pathway components [29–31].

Functionally, Hippo is composed by a regulatory module and a transcriptional module [32]. The first encompasses the kinases sterile 20-like kinase 1 and 2 (MST1/2) and large tumor suppressor 1 and 2 (LATS1/2), which require the adaptor proteins Salvador homologue 1 (SAV1), MOB kinase activator 1A and 1B (MOB1A/1B). The activation of the signaling cascade culminates in an inhibitory phosphorylation of two closely related oncoproteins: the transcriptional co-activator with PDZ-binding motif (TAZ) and the Yes-associated protein (YAP). When this occurs, TAZ/YAP are retained in the cytoplasm and/or excluded from the nucleus, and eventually undergo proteasomal degradation [22]. Defective activation of the regulatory module, or activation of other mechanisms that can directly tune TAZ/YAP, promotes the accumulation of Hippo transducers into the nucleus. Here, TEA domain-containing sequence-specific transcription factors (TEADs) serve as DNA-binding platforms for TAZ/YAP, mediating the transcription of target genes (e.g. CTGF, AXL CYR61, and ANKRD1) [22]. Thus, Hippo is a tumor-suppressor pathway essential for restraining the oncogenic functions elicited by TAZ/YAP.

In FBC, TAZ/YAP activation was tied to the retention/acquisition of cancer stem cell (CSCs) features [33, 34]. We and others demonstrated that TAZ sustains self-renewal, tumor-forming ability, chemoresistance and metastatic spread of breast CSC (BCSCs) [33, 34]. Our group has recently promoted translational studies aimed at characterizing this emerging BCSC pathway in cancer patients, with the goal of identifying potential prognostic and predictive biomarkers. Results from exploratory analyses in FBC patients provided the first clues that the expression of Hippo transducers may confer poorer clinical outcomes [35, 36].

Within the framework of an Italy-UK collaboration striving to provide novel molecular and clinical information on MBC, we herein investigated TAZ/YAP and their target CTGF in a large cohort of patients. Our goals included the following: i) describing the expression pattern of the Hippo transducers TAZ/YAP, ii) providing hints on their functional status by assessing CTGF as a readout for their activation, iii) providing information on their association with histological and molecular features (e.g. histotype, tumor grade, nodal status, hormone receptors, Ki-67), and iv) analyzing the impact of their expression on overall survival.

## RESULTS

**Figure 1:**
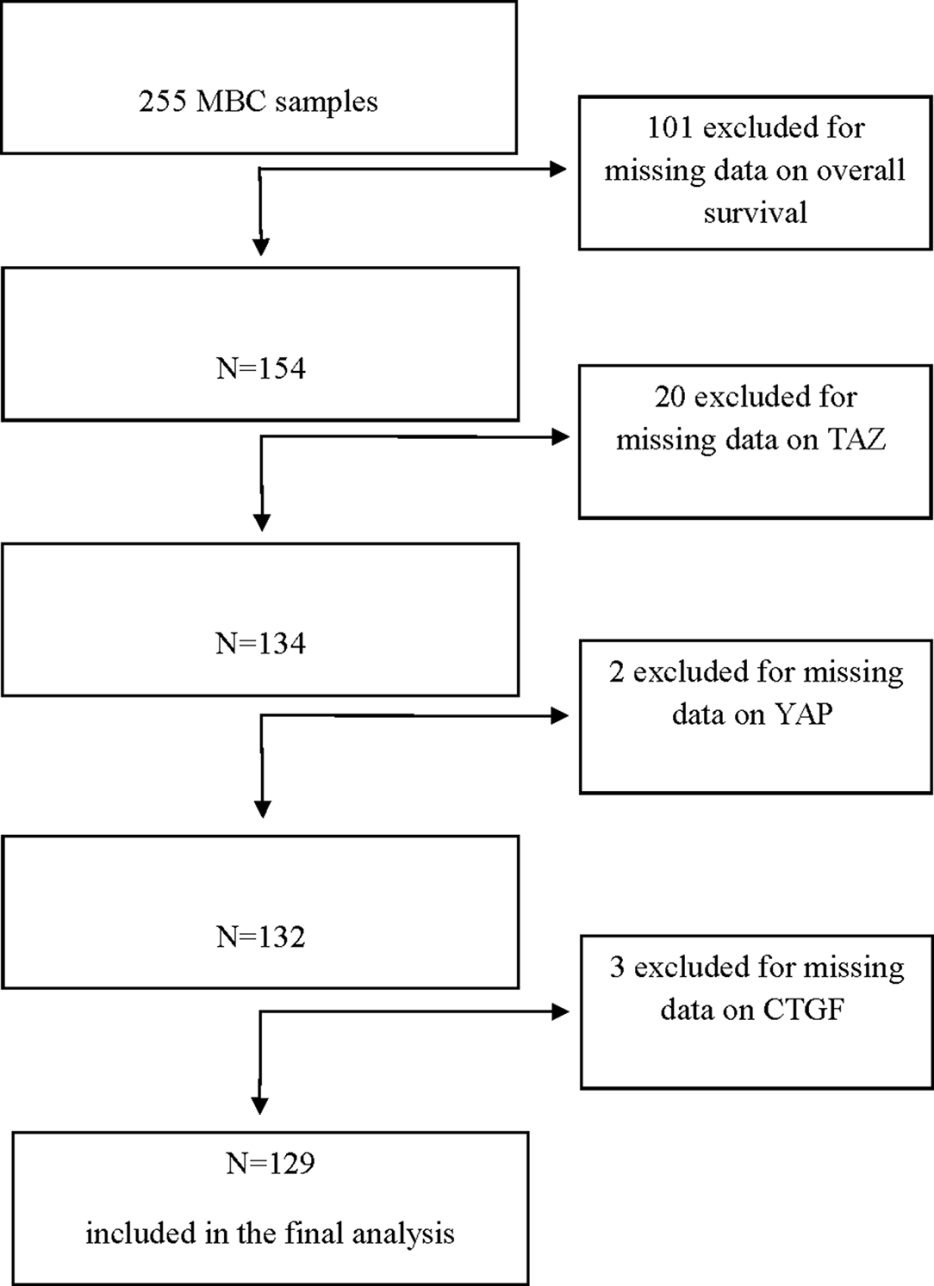
Flow diagram of the patients' selection process

For this study, 255 MBC patients were screened for the expression of TAZ, YAP and CTGF in their tumors. Upon molecular analyses, 129 patients were considered eligible (Figure 1). Baseline characteristics of these patients are summarized in Table 1. Representative immunohistochemical staining patterns are illustrated in Supplementary Figure 1.

**Table 1:** Baseline characteristics of the study participants (*N* = 129)

Characteristics	*N* (%)
Age at diagnosis^*^	
Median (min-max) [IQ range]	67.5 (34–88) [59–75]
**Histology**	
IDC/ILC	108 (83.7)
Other	21 (16.3)
**Grade**	
G1-2	67 (51.9)
G3	62 (48.1)
**Nodal status**	
Negative	39 (30.2)
Positive	53 (41.1)
Unknown	37 (28.7)
**Hormone receptors**	
ER^+^/PgR^+^	109 (84.5)
Other	20 (15.5)
**Ki-67**	
Low (< 14%)	73 (56.6)
High (≥ 14%)	56 (43.4)
**TAZ**	
0	44 (34.1)
Cytoplasm	56 (43.4)
Nucleus	3 (2.3)
Nucleus/Cytoplasm	26 (20.2)
**YAP**	
0	13 (10.1)
Cytoplasm	98 (76.0)
Nucleus	0 (0.0)
Nucleus/Cytoplasm	18 (14.0)
**CTGF**	
Neg	77 (59.7)
Pos	52 (40.3)

* computed in 108 patients.

Abbreviations: IDC: invasive ductal carcinoma, ILC: invasive lobular carcinoma.

**Figure 2:**
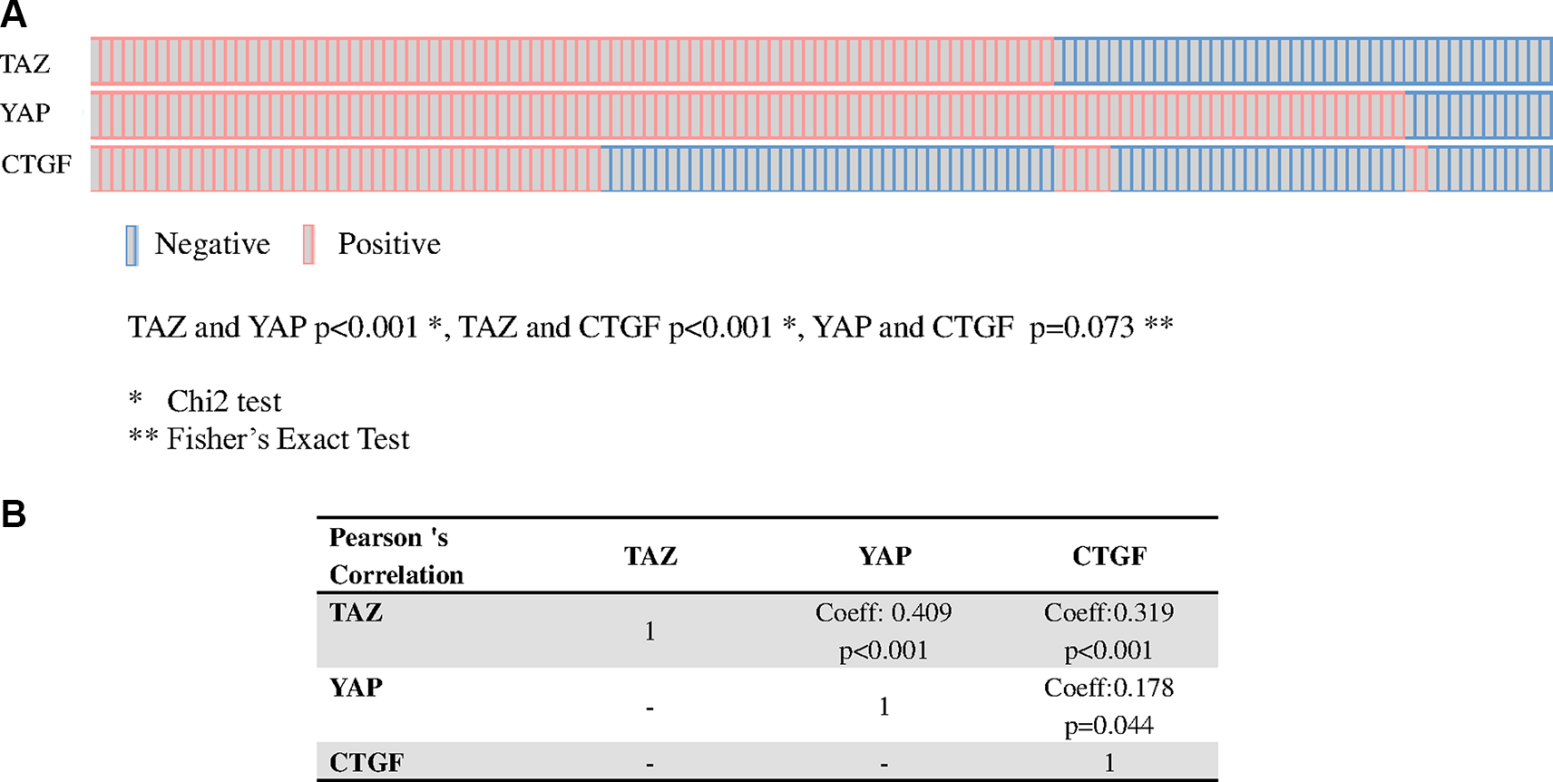
Relationship between TAZ, YAP and CTGF Association (OncoPrints in **panel A**) and correlation (**panel B**) between TAZ, YAP and CTGF in 129 male breast cancer samples.

We observed a positive association between TAZ and YAP and between TAZ and CTGF, and a suggestion for an association between YAP and CTGF (TAZ and YAP *p* < 0.001; TAZ and CTGF *p* < 0.001; YAP and CTGF *p* = 0.073, Figure 2, panel A). Moreover, a significant correlation between TAZ and CTGF and YAP and CTGF was seen in terms of tumor-expressing cells (Figure 2, panel B). On this basis, samples were considered to have active TAZ/YAP-driven gene transcription when they co-expressed TAZ and CTGF (TAZ/CTGF) or YAP and CTGF (YAP/CTGF).

**Figure 3:**
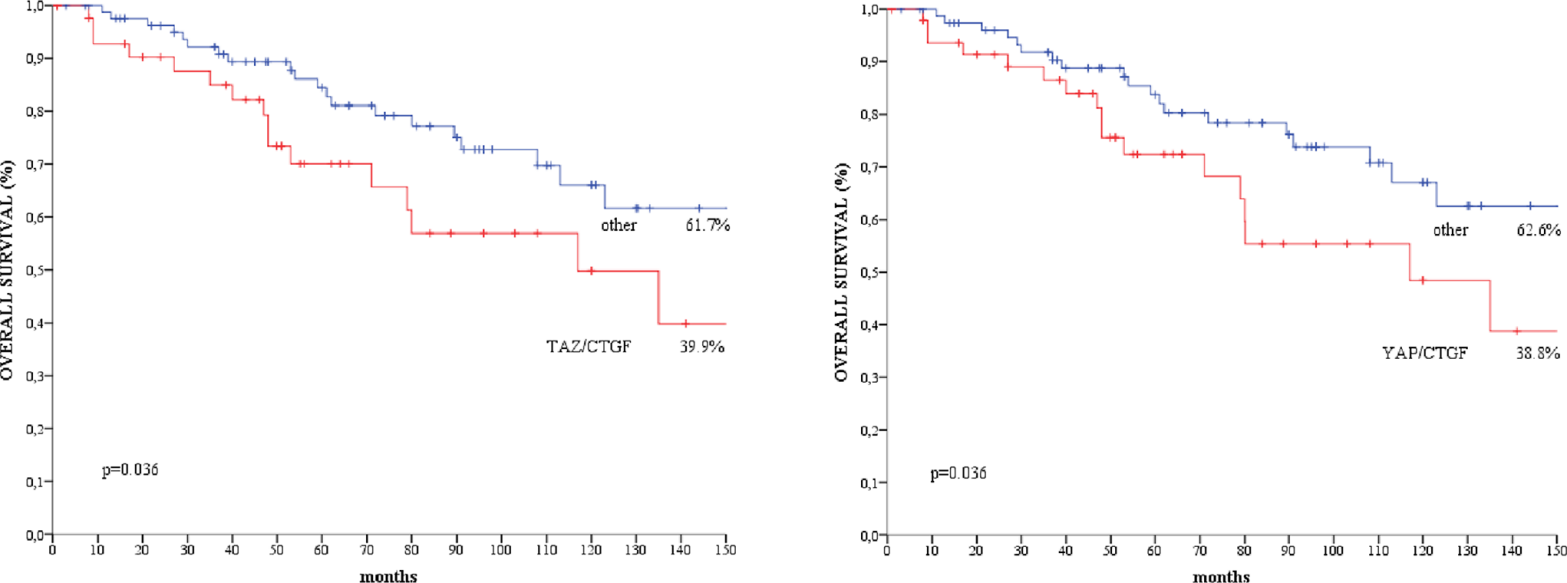
Kaplan-Meier survival curves of overall survival regarding (**A**) TAZ/CTGF and (**B**) YAP/CTGF in the entire study population (*N* = 129).

**Figure 4:**
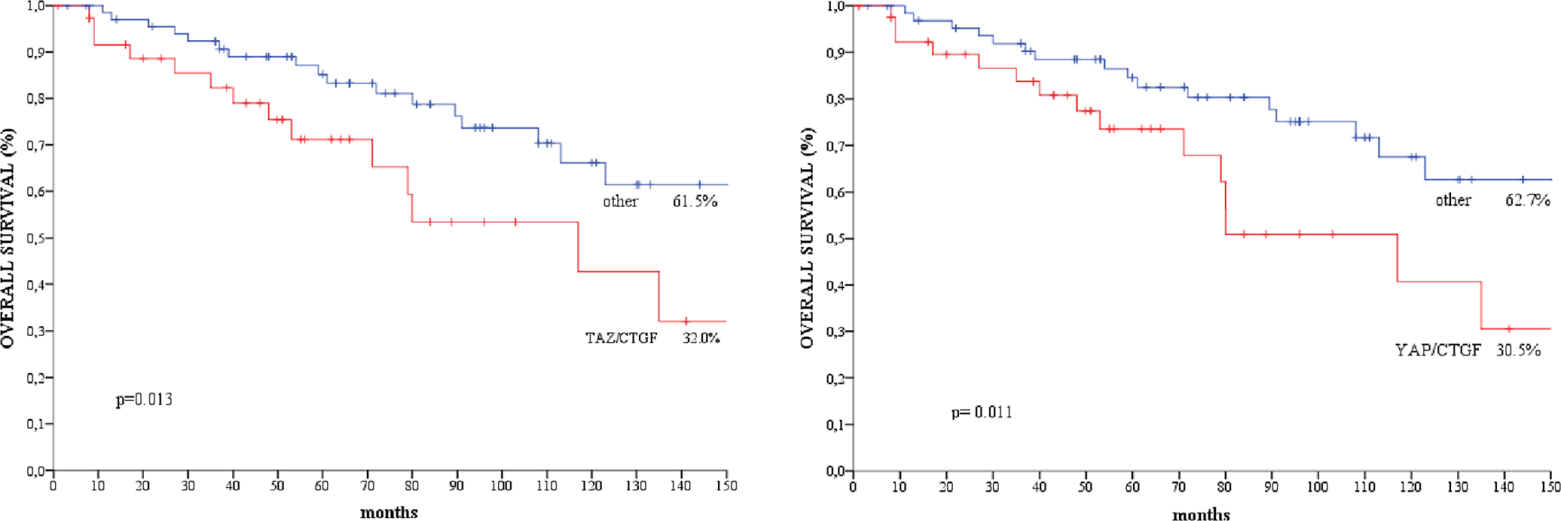
Kaplan-Meier survival curves of overall survival regarding (**A**) TAZ/CTGF and (**B**) YAP/CTGF in patients with IDC/ILC (*N* = 108).

We did not observe any significant association between the TAZ/CTGF and YAP/CTGF phenotypes and clinical-molecular features (Supplementary Table 1). At a median follow-up of 183 months, 37 deaths occurred. Patients with TAZ/CTGF and YAP/CTGF tumors had significant poorer overall survival compared with their negative counterparts (log rank *p* = 0.036 for both, Figure 3), and comparable results were obtained when considering triple-positive tumors (TAZ/YAP/CTGF, log rank *p* = 0.036, data available upon request). These findings were further confirmed when analyzing the 108 patients with invasive ductal carcinoma (IDC) or invasive lobular carcinoma (ILC) (log rank *p* = 0.013 and *p* = 0.011 for TAZ/CTGF and YAP/CTGF, respectively, as shown in Figure 4).

**Figure 5:**
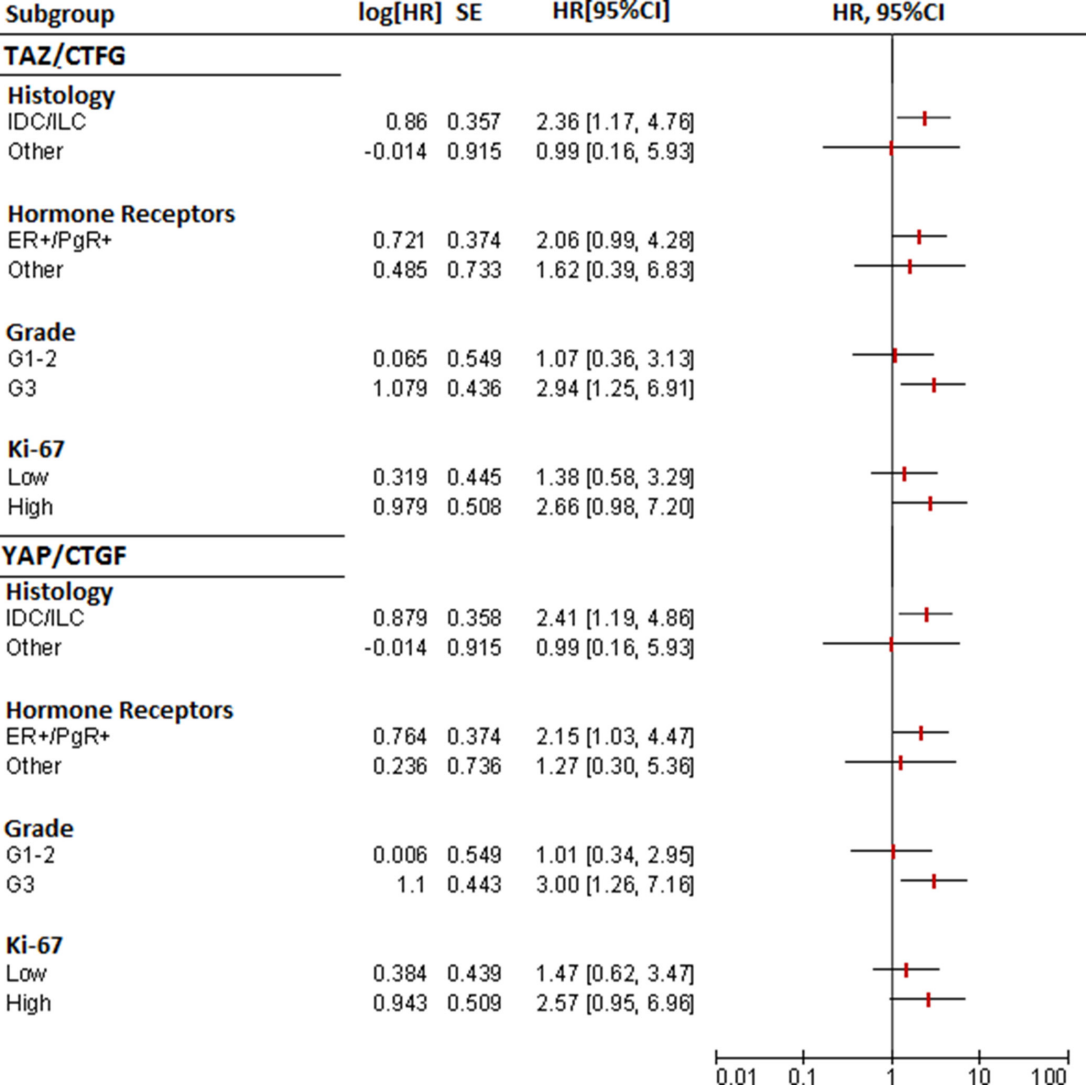
Forest plots for subgroup analysis of overall survival (TAZ/CTGF and YAP/CTGF models)

In subgroup analyses, the TAZ/CTGF and YAP/CTGF phenotypes were associated with an increased risk of death also in patients with ER+/PgR+ tumors, G3 tumors, and tumors with elevated Ki-67 levels (≥ 14) (Figure 5).

Multivariate Cox regression models, presented in Table 2, confirmed that patients with TAZ/CTGF and YAP/CTGF tumors were at increased risk of death (HR 2.03, 95% CI: 1.06–3.90, *p* = 0.033; and HR 2.00, 95% CI: 1.04–3.84, *p* = 0.037, respectively). This association was maintained even when adjusting by clinical-molecular variables that did not test significant at the univariate assessment (TAZ/CTGF: HR 2.10, 95% CI: 1.08–4.07, *p* = 0.028. YAP/CTGF: HR 2.10, 95% CI: 1.07–4.09, *p* = 0.030, Table 2). In a sensitivity analysis carried out by excluding uncommon histotypes (*N* = 21), TAZ/CTGF and YAP/CTGF were the only variables that tested significant in multivariate Cox regression models (HR 2.34, 95% CI: 1.16–4.73, *p* = 0.018, and HR 2.36, 95% CI: 1.17–4.77, *p* = 0.017, respectively, Table 3). Finally, nearly comparable results were obtained in the subpopulation of MBC patients with available nodal status (*N* = 92) (Supplementary Table 2).

**Table 2:** Univariate and multivariate Cox regression models of disease-related features and overall survival (*N* = 129)

		Univariate Cox		Multivariate Cox							
		Regression model		Regression model^§^				Regression model^#^			
		HR (95% CI)	*p*-value	HR (95% CI)	*p*-value	HR (95% CI)	*p*-value	HR (95% CI)	*p*-value	HR (95% CI)	*p*-value
**Histology**	IDC/ILC vs other	1.20(0.47–3.08)	0.709					1.12(0.42–2.99)	0.817	1.13(0.42–3.00)	0.810
**Grade**	G3 vs G1-2	2.01(1,03–3.93)	0.042	2.06(1.05–4.05)	0.035	2.04(1.04–4.00)	0.039	1.93(0.94–3.93)	0.072	1.89(0.92–3.85)	0.081
**Hormone receptors**	ER^+^/PgR^+^ vs other	0.77(0.35–1.70)	0.516					0.80(0.36–1.78)	0.578	0.79(0.35–1.78)	0.571
**Ki-67**	High vs Low	1.16(0.61–2.24)	0.651					1.13(0.58–2.24)	0.072	1.18(0.60–2.34)	0.637
**TAZ/CTGF**	TAZ/CTGF vs other	1.98(1.03–3.78)	0.040	2.03(1.06–3.90)	0.033			2.10(1.08–4.07)	0.028		
**YAP/CTGF**	YAP/CTGF vs other	1.97(1.03–3.77)	0.040			2.00(1.04–3.84)	0.037			2.10(1.07–4.09)	0.030

§ Backward stepwise exclusion.

# Adjusted for: Histology, Grade, Hormone receptor status, and Ki-67.

**Table 3:** Univariate and multivariate Cox regression models of disease-related features and overall survival in MBC patients with IDC/ILC (*N* = 108)

		Univariate Cox		Multivariate Cox							
		Regression model		Regression model^§^				Regression model^#^			
		HR (95% CI)	*p*-value	HR (95% CI)	*p*-value	HR (95% CI)	*p*-value	HR (95% CI)	*p*-value	HR (95% CI)	*p*-value
**Grade**	G3 vs G1-2	1.98 (0.95–4.14)	0.069	1.96 (0.94–4.09)	0.075	1.92 (0.92–4.03)	0.082	1.82 (0.84–3.94)	0.132	1.76 (0.81–3.84)	0.154
**Hormone receptors**	ER^+^/PgR^+^ vs other	0.63 (0.28–1.42)	0.266					0.66 (0.28–1.53)	0.328	0.65 (0.28–1.53)	0.327
**Ki-67**	High vs Low	1.00 (0.49–2.03)	0.998					1.03 (0.49–2.17)	0.937	1.09 (0.51–2.31)	0.825
**TAZ/CTGF**	TAZ/CTGF vs other	2.36 (1.17–4.76)	0.016	2.34 (1.16–4.73)	0.018			2–40 (1.18–4.90)	0.016		
**YAP/CTGF**	YAP/CTGF vs other	2.41 (1.20–4.86)	0.014			2.36 (1.17–4.77)	0.017			2.44 (1.18–5.04)	0.016

§ Backward stepwise exclusion.

# Adjusted for: Histology, Grade, Hormone receptor status, and Ki-67.

## DISCUSSION

In this study, we investigated the expression of key components of the Hippo transcriptional module in a large cohort of MBC patients with available survival data. Our results suggest that MBC patients whose tumors co-express TAZ, or YAP, and CTGF may have inferior survival compared with their negative counterparts. To our knowledge, this is the first study pointing to the Hippo pathway in MBC. Even though our results are hypothesis-generating, we were able to apply preclinical information related to an emerging oncogenic avenue to a rare disease such as MBC, and with a clear focus on clinical outcomes.

We acknowledge that our results should be viewed with caution in consideration of the retrospective nature of this study. Moreover, some potential limitations deserve mention. First, despite our best efforts, we were unable to gather all the necessary information related to systemic anticancer treatments. Second, in this series 37 events were recorded. Fourteen events were MBC-related deaths, but we ignore the cause of the remaining 23 deaths (62% of the total). This hindered the analysis of cancer specific mortality in an elderly patient population. Unfortunately, we are unable to address this issue. However, the TAZ/CTGF and YAP/CTGF phenotypes were predictive of overall survival in: i) patients whose tumors displayed aggressive molecular features (i.e. high grade and elevated Ki-67 levels), and ii) patients with the most common form of MBC (i.e. ER+ and PgR+ tumors, IDC). These characteristics delineate the prototype of MBC patients who usually receive adjuvant therapy, and eventually will experience disease recurrence. Consistently, patients with TAZ/CTGF- and YAP/CTGF-positive tumors had significant shorter disease-free survival (DFS) compared with their negative counterparts (log rank *p* = 0.038 and *p* = 0.046, respectively, reported in Supplementary Figure 2), even though missing information pertinent to the events considered in the definition of DFS (local recurrence, distant recurrence, death from any cause) needs to be considered. More importantly, multivariate Cox regression models suggested that the TAZ/CTGF and YAP/CTGF variables were independent predictors of survival.

Despite these limitations, our study provided some intriguing clues. We have already discussed some strategies for interpreting TAZ/YAP expression [21]. Nuclear localization is often considered as a proxy of TAZ/YAP activation. Nevertheless, TAZ/YAP activity supposedly oscillates over time depending on specific tissue contexts [22]. Different observations account for that. First, somatic mutations in Hippo pathway components potentially leading to TAZ/YAP constitutional activity are uncommon [21]. For instance, neurofibromin 2 (NF2, also known as Merlin), which is known to be mutationally inactivated in some cancers [32], is rarely mutated in FBC [37] and initial genetic characterization of MBC did not unveil any alteration in core Hippo pathway components [38]. Thus, defective control of TAZ/YAP is probably driven by functional cues, rather than by genetic events. This is not surprising when considering the nature of key regulatory branches controlling their nuclear shuttling, such as cell-cell adhesion mechanisms, apical-basal polarity factors, and mechanical forces (mechanotransduction) imposed on cancer cells by the extracellular matrix (ECM) and cell density [34, 39–41]. These stimuli feed TAZ/YAP activation in a context-dependent manner on the basis, for instance, of the topographic localization of cells within a tumor. The same considerations can be extended to hypoxic regions, when considering the reciprocal interaction between hypoxia-inducible factor 1 (HIF-1) and TAZ in BCSC models [42, 43]. Second, TAZ/YAP require the interaction with other factors, including TEAD, SMAD and RUNX proteins, for promoting the transcription of target genes. Defective interaction with, or activity of, these partners hampers gene transcription independently on whether TAZ/YAP localize to the nucleus [44]. Seminal evidence reported frequent RUNX1 deletions and MLL3 mutations in MBC [38], and both these factors have been associated with TAZ/YAP-mediated gene transcription [45, 46].

To overcome this complexity, we decided to concomitantly analyze an established target of TAZ/YAP. We chose CTGF for two reasons. First, CTGF is widely exploited for monitoring TAZ/YAP activation upon their forced overexpression, or knockout, in cellular models. Second, albeit TAZ and YAP are closely related proteins, the spectrum of up-regulated genes after their overexpression is partly different [47]. Nevertheless, CTGF ranked among top commonly up-regulated genes [47]. Based on this premise, and prompted by the co-expression pattern, we opted for the TAZ/CTGF and YAP/CTGF models for defining positive and negative cases.

Next, in FBC activation of TAZ has been linked to BCSCs through a multiplicity of mechanisms [33, 34, 42, 43, 48, 49]. The first link between Hippo and BCSCs stemmed from the over-representation of a TAZ/YAP signature in G3 versus G1 tumors [34]. Consistently, poorly differentiated FBC are supposed to be enriched for CSCs [50]. We did not appreciate any clear association between TAZ/CTGF and YAP/CTGF phenotypes and tumor grade in the entire cohort. Nevertheless, when we exclusively considered G1 and G3 tumors, TAZ/CTGF and YAP/CTGF were significantly associated with poor differentiation (*p* = 0.006 and *p* = 0.002, respectively, data available upon request). Moreover, in subgroup analysis, the expression of TAZ/CTGF and YAP/CTGF conferred poorer overall survival in patients with G3 tumors, but not in those with G1-2 tumors. In the entire population (*N* = 129), higher tumor grade seemed to be also associated with shorter survival, and this is consistent with an independent study that analyzed survival outcomes of ~3.000 MBC patients [51]. A plausible hypothesis is that TAZ/YAP activity may be also necessary for maintenance/amplification of the CSC compartment in MBC. Considering the unavailability of MBC cellular models for preclinical studies and the growing interest surrounding CSC-related biomarkers for predicting therapeutic outcomes, we are striving to establish MBC patient-derived tumor-initiating cells for functional characterization and mechanistic studies.

Another aspect that deserves mention refers to the metabolic control of TAZ/YAP and its connection with hormone receptor pathways. As aforementioned, a variety of stimuli that either encourage or restrain TAZ/YAP activity intersect the Hippo cascade. The mevalonate pathway was recently described as an important regulator of TAZ/YAP [52]. Geranylgeranyl pyrophosphate produced in the mevalonate cascade is required for correct membrane tethering of Rho-GTPases which, in turn, act as positive regulators of TAZ/YAP. Consistently, inhibition of HMG-CoA reductase, the rate-limiting enzyme of the mevalonate pathway, achieved with statins hindered the metabolic control of TAZ/YAP via altered prenylation of Rho GTPases [52]. Beyond protein prenylation, the mevalonate pathway is central for steroid and cholesterol biosynthesis. We hypothesized that HMG-CoA reductase may be a key intracellular node in MBC, and a nexus between TAZ/YAP and hormone receptors. Indeed, activation of the mevalonate cascade may influence the hormonal background that nourishes MBC cells, an hypothesis also supported by the link existing between obesity and MBC [3], while concomitantly activating TAZ/YAP via Rho GTPases. In the same cohort herein presented, we also carried out HMG-CoA reductase assessment. Our preliminary, still unpublished results indicate that HMG-CoA reductase expression is positively associated with both hormone receptors and Hippo transducers. This allowed us to envision an oncogenic endocrine-metabolic background, possibly acting at both the systemic and local level, where the mevalonate pathway, endocrine stimuli and aberrant TAZ/YAP-driven gene transcription are tightly connected and cooperate in establishing an oncogenic network feeding MBC cells. Not surprisingly, recent preclinical evidence elucidated a positive estrogen-mediated control of TAZ/YAP operated by the G protein-coupled estrogen receptor (GPER) via the Gαq-11, PLCβ/PKC, and Rho/ROCK signaling pathways [53]. Based on the established association between obesity and MBC, along with the molecular data gathered on the plausible interconnection among the determinants of interest, e.g., Hippo transducers, mevalonate pathway and hormone receptors, we envision a role for statins in the treatment of MBC patients with specific molecular characteristics. To this end, observational studies evaluating the impact of statin use on survival outcomes, ideally upon assessment of specific markers suggestive of an endocrine-metabolic-driven disease, are warranted.

Overall, our data suggested that the co-expression of the Hippo transducers TAZ/YAP and CTGF may be an adverse prognostic factor in MBC. Further research on this topic, envisioning a wider pathway analysis, is ongoing and will better delineate the contribution of altered Hippo signaling on MBC biology.

## MATERIALS AND METHODS

### Study participants and procedures

For this retrospective study, samples from 255 histologically confirmed MBC patients, previously characterized for common clinical-molecular features (histology, grade, hormone receptors, Ki-67) [7], were immunohistochemically analyzed for the expression of TAZ, YAP and CTGF. Patients did not receive any therapy before surgery. Adjuvant therapy mostly consisted in tamoxifen, but extensive data on systemic anticancer therapies were not available [7]. For this study, we applied the following eligibility criteria: i) complete data on TAZ, YAP and CTGF, and ii) availability of overall survival data. Of the 255 patients evaluated, 129 were considered eligible for this study. Nodal status was available for 92 patients. This subset of patients was separately analyzed. Overall survival (OS) was defined as the time from diagnosis until death from any cause.

Tissue microarrays (TMAs) were built by using 3 × 0.6 mm tissue cores per case obtained from formalin-fixed paraffin-embedded (FFPE) material, as already detailed [7]. The immunohistochemical assessment of TAZ, YAP and CTGF was performed in FFPE tissues using the monoclonal antibody (MoAb) anti-TAZ (M2-616, BD Pharmingen, San Jose, CA, USA) at the dilution of 1:400, the MoAb anti-YAP (H-9, Santa Cruz Biotechnology, Santa Cruz, CA, USA) at the dilution of 1:200, and the polyclonal antibody anti-CTGF (HPA031074, Sigma-Aldrich, Saint Louis, MO) at the dilution of 1:50.

TAZ, YAP and CTGF were graded on a four-grade scale (0: negative, 1+: weak, 2+: moderate, 3+: strong). TAZ and YAP were considered positive if ≥ 10% of neoplastic cells exhibited a distinct nuclear and/or cytoplasmic immunoreactivity of any intensity. CTGF was considered positive when ≥ 10% of neoplastic cells exhibited a distinct cytoplasmic immunoreactivity of any intensity. Two investigators (ADB and MM) blinded to treatment outcome evaluated immunoreactivity.

This retrospective study was conducted in accordance with the Declaration of Helsinki and approved by the Institutional Review Board of the “Regina Elena” National Cancer Institute of Rome, the coordinating centre, and by the Leeds (East) Research Ethics Committee (06/Q1205/156). Informed consent was not required, as already specified [7]. The study adheres to the REporting recommendations for tumor MARKer prognostic studies (REMARK) criteria.

### Statistical analysis

Descriptive statistics were used to summarize the characteristics of the study participants. The relationship between categorical variables was assessed with the Pearson's Chi-squared test of independence (2-tailed). The Fisher Exact test was used, when appropriate, depending upon the size of the groups compared. The Pearson's correlation coefficient was used to investigate the correlation between TAZ, YAP and CTGF. The Kaplan-Meier method was used for estimating survival curves, which were compared by log-rank test. In order to identify independent predictors of survival, multivariate Cox proportional hazard models were built using stepwise regression (backward elimination). The related estimates were reported as Hazard Ratios (HR) and 95% Confident Interval (CI). Cox models were also generated by adjusting for clinical-molecular variables that may impact the investigated outcome. For the treatment of missing values (histology: 1/129, tumor grade: 3/129, hormone receptors: 2/129, Ki-67 levels: 4/129), random hot deck (RHD) imputation was used [54]. Pools of potential donors were identified by matching for a set of auxiliary variables (histology, tumor grade, hormone receptor status and Ki-67, depending upon the nature of the missing variable). Afterwards, a donor was randomly selected and the value of interest assigned to the recipient. We considered statistically significant *p* values less than 0.05. Statistical analyses were carried out using SPSS software (SPSS version 21, SPSS Inc., Chicago, IL, USA).

## SUPPLEMENTARY MATERIALS FIGURES AND TABLES


