# Supplementary material for: The Hippo transducers TAZ/YAP and their target CTGF in male breast cancer
Source: Oncotarget. 2016 May 27;7(28):43188–98. doi: 10.18632/oncotarget.9668 (PMC5190017; doi:10.18632/oncotarget.9668)
Supplement: Supplementary file 1 [file oncotarget-07-43188-s001.pdf]

The Hippo transducers TAZ/YAP and their target CTGF in male breast cancer

Supplementary Materials

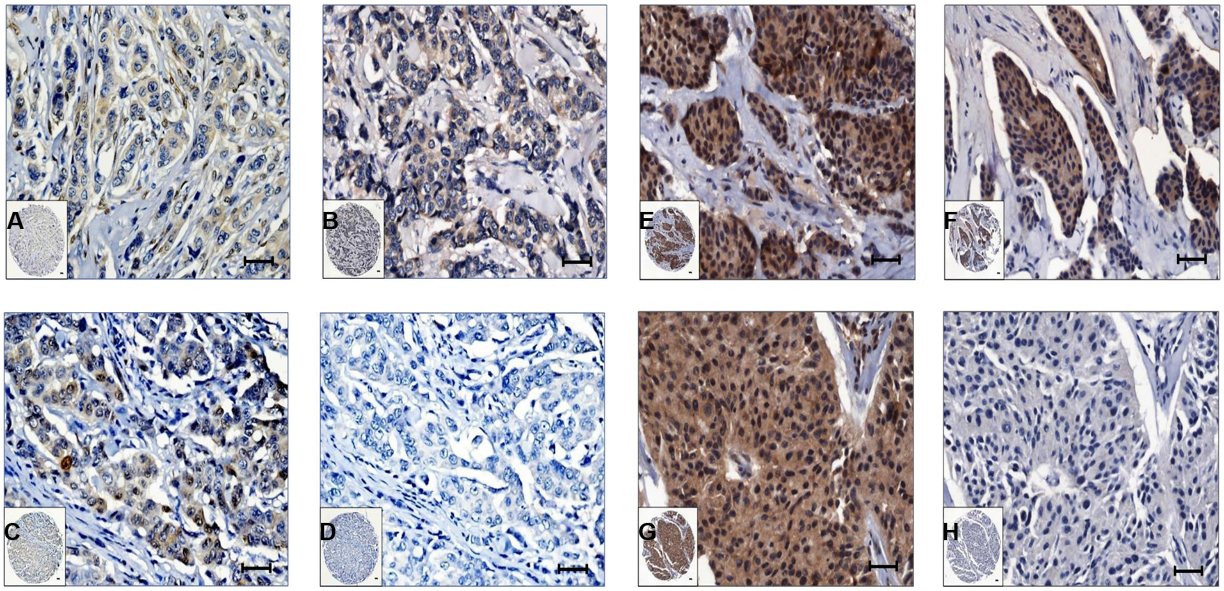

Supplementary Figure S1: Representative examples of immunohistochemical expression of TAZ, YAP and CTGF in four male breast cancer patients. Panels a-b show a sample with expression of TAZ (**A**) and cytoplasmic CTGF immunoreactivity (**B**). In panels (**C–D**), a tumor case with expression of TAZ (C), but negative for CTGF (D). Panels e-f show a sample with expression of YAP (**E**) and cytoplasmic CTGF immunoreactivity (**F**). Panels g-h show a tumor case with expression of YAP (**G**), but negative for CTGF (**H**). Scale bar 30 µm. Inset magnification × 10.

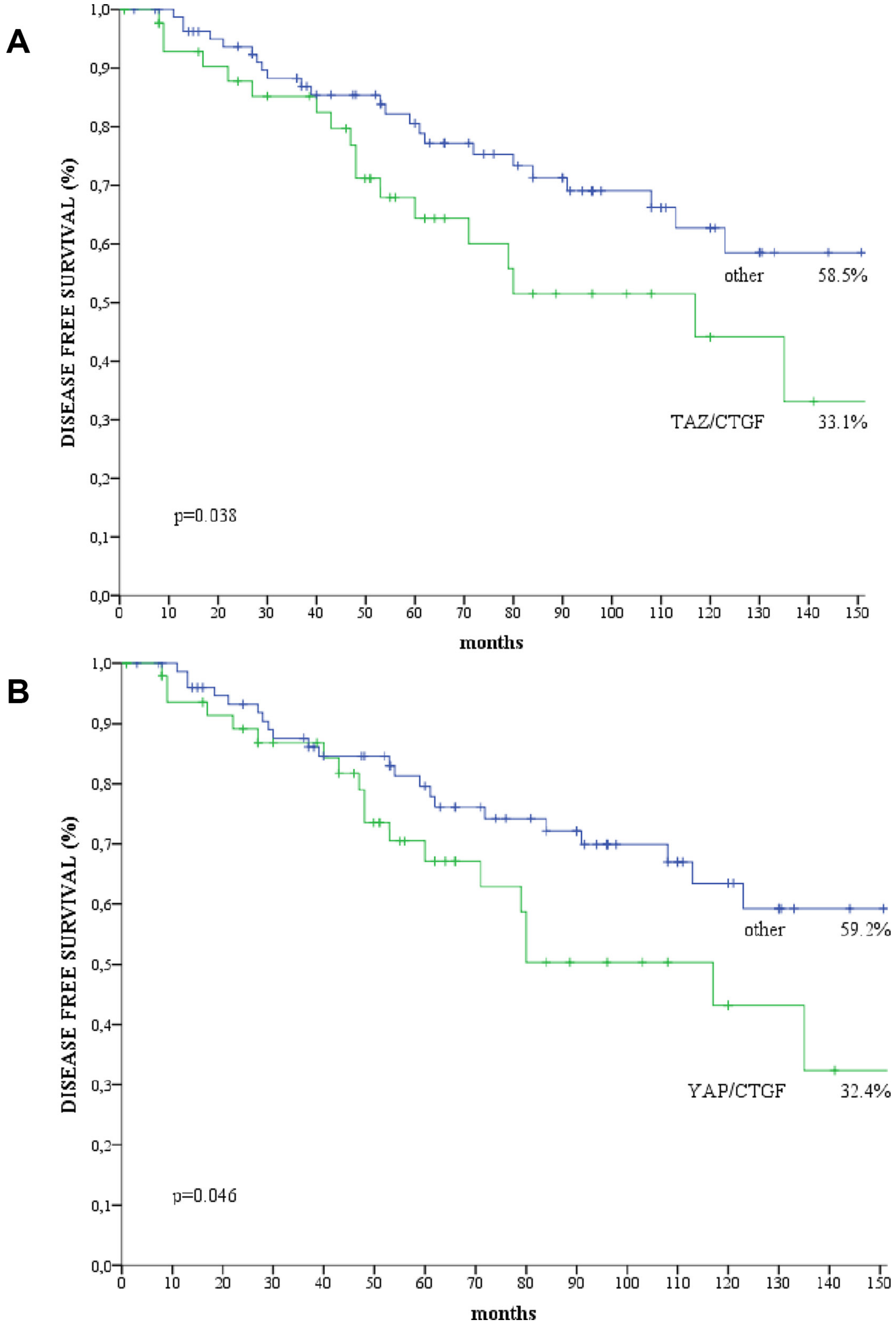

Supplementary Figure S2: Kaplan-Meier survival curves of disease-free survival regarding: (A) TAZ/CTGF and (B) YAP/CTGF in the entire study population (*N* = 129).

Supplementary Table S1: Association between TAZ/CTGF and YAP/CTGF phenotypes and clinical-molecular variables

	TAZ/CTGF		Chi² Test
	Other	TAZ/CTGF	*p*-value
	N (%)	*N* (%)	
**Histology**			
IDC/ILC	69 (63.9)	39 (36.1)	0.507
Other	15 (71.4)	6 (28.6)	
**Grade**			
G1-2	48 (71.6)	19 (28.4)	0.106
G3	36 (56.1)	26 (41.9)	
**Nodal status**			
Negative	22 (56.4)	17 (43.6)	0.258
Positive	36 (67.9)	17 (32.1)	
**Hormone receptors**			
ER^+/PgR^+	70 (64.2)	39 (35.8)	0.618
Other	14 (70.0)	6 (30.0)	
**Ki-67**			
Low (< 14%)	47 (64.4)	26 (35.6)	0.842
High (≥ 14%)	37 (66.1)	19 (33.9)	

	YAP/CTGF		Chi² Test
	Other	YAP/CTGF	*p*-value
	N (%)	*N* (%)	
**Histology**			
IDC/ILC	66 (61.1)	42 (38.9)	0.946
Other	13 (61,9)	8 (38.1)	
**Grade**			
G1-2	46 (68.7)	21 (31.3)	0.072
G3	33 (53.2)	29 (46.8)	
**Nodal Status**			
Negative	20 (51.3)	19 (48.7)	0.154
Positive	35 (66.0)	18 (34.0)	
**Hormone receptors**			
ER^+/PgR^+	66 (60.6)	43 (39.4)	0.707
Other	13 (65.0)	7 (35.0)	
**Ki-67**			
Low (< 14%)	43 (58.9)	30 (41.1)	0.534
High (≥ 14%)	36 (64.3)	20 (35.7)	

Supplementary Table S2: Univariate and multivariate Cox regression models in patients with available nodal status ($N = 92$)

		Univariate Cox		Multivariate Cox							
		Regression model		Regression model[§]				Regression model[#]			
		HR (95% CI)	*p*-value	HR (95% CI)	*p*-value	HR (95% CI)	*p*-value	HR (95% CI)	*p*-value	HR (95% CI)	*p*-value
**Histology**	IDC/ILC vs other	0.83 (0.28–2.43)	0.729					0.86 (0.28–2.63)	0.793	0.90 (0.29–2.76)	0.849
**Grade**	G3 vs G1-2	1.78 (0.80–3.96)	0.161	2.02 (0.89–4.58)	0.093	1.97 (0.87–4.45)	0.103	1.96 (0.84–4.59)	0.122	1.89 (0.81–4.42)	0.143
**Nodal Status**	Pos vs Neg	0.78 (0.36–1.68)	0.525					0.72 (0.32–1.60)	0.416	0.75 (0.33–1.67)	0.473
**Hormone receptors**	ER^+/ PgR^+ vs other	0.75 (0.29–1.89)	0.537					0.64 (0.24–1.70)	0.372	0.62 (0.23–1.64)	0.334
**Ki-67**	High vs Low	1.53 (0.70–3.37)	0.288					1.62 (0.71–3.72)	0.255	1.69 (0.74–3.88)	0.143
**TAZ/ CTGF**	TAZ/ CTGF vs other	1.98 (0.91–4.29)	0.085	2.21 (1.00–4.89)	0.049			2.15 (0.97–4.79)	0.061		
**YAP/ CTGF**	YAP/ CTGF vs other	2.11 (0.97–4.59)	0.061			2.29 (1.04–5.05)	0.039			2.30 (1.02–5.20)	0.045

[§]Backward stepwise exclusion. [#] Adjusted for: Histology, Grade, Nodal Status, Hormone receptor status, and Ki-67.
